# Treatment of colon cancer in a patient with systemic lupus erythematosus: a case report

**DOI:** 10.1186/s12885-018-4864-x

**Published:** 2018-10-10

**Authors:** Junfeng Shi, Jianquan Fei, Qingqing Yi, Lijuan Shen, Boshun Wan, Yueyu Chen, Qing Chang

**Affiliations:** 1 Shanghai Key Laboratory for Molecular Imaging, Shanghai University of Medicine and Health Sciences, Pudong New Area, Shanghai, 201318 China; 2 Department of General Surgery, Jiading District Central Hospital Affiliated Shanghai University of Medicine and Health Sciences, Shanghai, 201800 China; 3 Clinical Research Center, Jiading District Central Hospital Affiliated Shanghai University of Medicine and Health Sciences, Shanghai, 201800 China; 4 Department of Clinical Laboratory, Jiading District Central Hospital Affiliated Shanghai University of Medicine and Health Sciences, Shanghai, 201800 China

**Keywords:** Systemic lupus erythematosus, Colon cancer, Radical resection, Prednisone, Methylprednisolone

## Abstract

**Background:**

Gastrointestinal symptoms occur in approximately 50% of patients with systemic lupus erythematous with low specificity. Although it is well established that colon cancer is one of the many gastrointestinal manifestations associated with systemic lupus erythematous, the diagnosis and treatment remains complex due to adrenal insufficiency symptoms.

**Case presentation:**

A 43-year-old Chinese woman with a five-year history of systemic lupus erythematous was diagnosed with colon cancer based on imaging test. A radical bowel resection was performed successfully. To avoid serious complications during surgery, prednisone was replaced with methylprednisolone therefore avoiding adrenal insufficiency. The patient was subsequently treated with mFOLFOX6 chemotherapy and recovered well.

**Conclusion:**

There is a lack of reports for treatment of colon cancer in patients with systemic lupus erythematous. This report provides an effective way to diagnose colon cancer in patients with systemic lupus erythematous and illustrates a successful therapy strategy for this complex medical condition.

## Background

Systemic lupus erythematous (SLE) is an autoimmune disease with multi-organ involvement and often occurs in young women. Although SLE has the potential to affect almost all organs [1], including the gastrointestinal system, the essential examination of the gastrointestinal system on patients with SLE is often omitted [2].

In this report, we described an unusual case of a female patient with both SLE and colon cancer, and discussed the difficulties faced during the treatment of this patient.

## Case presentation

### Case report

A 43-year-old Chinese woman was diagnosed with SLE 5 years ago, and has been receiving ongoing treatment with prednisone and omeprazole orally. Starting from 2 years ago, the patient had difficult defecation and watery stools with left lower abdominal pain, usually half an hour postprandially. The symptoms had become progressively worse over the previous 2 months, and the patient was referred to our hospital.

Her physical examination on admission was normal except for a palpable lower abdominal mass, about four cube centimeters. Laboratory data displayed a high level of globulin, elevated D-Dimer level and weakly positive fecal occult blood test (Table 1). The serum levels of tumor biomarkers of colon cancer, carcinoembryonic antigen and CA19–9, were normal (Table 1).

**Table 1:** Laboratory data

	Pre-surgery	Post-surgery (44d)	Units
Red Blood Cell	3.89 × 1012	3.72 × 1012	/L
Hemoglobin	118	113	g/L
White Blood Cell	6.40 × 109	4.70 × 109	/L
Granulocytes	76.70	60.30	%
Albumin	40.60	39.60	g/L
Globulin	42.30	34.60	g/L
Albumin/Globulin	1.0	1.1	
D-Dimer	2.02	1.58	mg/L
Fecal Occult Blood Test	W+	–	
Carcinoembryonic Antigen	< 0.20	0.384	ng/ml
CA19–9	2.86	4.36	U/ml

**Fig. 1:**
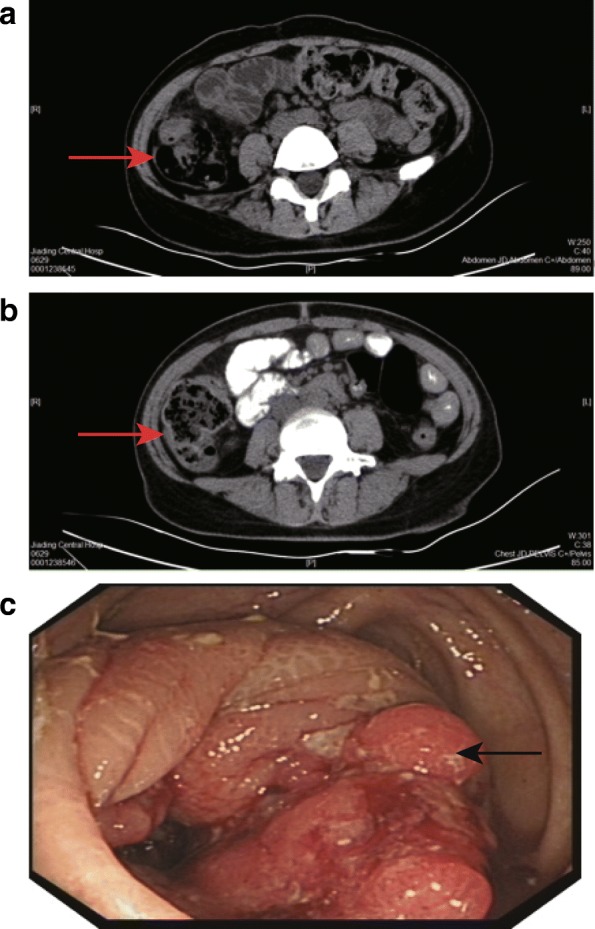
CT scan and colonoscopy showed wall thickening and a space-occupying lesion in the colon of the patient. **a**, upper abdominal CT scan showed wall thickening (red arrow) in the ascending colon; **b**, lower abdominal enhanced CT scan revealed wall thickening (red arrow) in the proximal ascending colon, distal cecum and ileum; **c**, a colonoscopy displayed a space-occupying lesion (black arrow) in the ascending colon. CT, computed tomography

Upper abdominal computed tomography (CT) scan showed that wall thickening partly occurred in the ascending colon, indicating a tumor lesion (Fig. 1a). Lower abdominal enhanced CT scan revealed wall thickening in the proximal ascending colon, distal cecum and ileum, which suggests a tumor lesion and peri-intestinal infiltration (Fig. 1b). A colonoscopy displayed a space-occupying lesion in the ascending colon (Fig. 1c).

Based on the evaluation mentioned above, colon cancer with SLE was suspected pending the biopsy results. A radical bowel resection was considered as a preferred strategy. However, the patient had taken prednisone and omeprazole per os for an extended time period, which increases the susceptibility to possible complications, such as infection, gastrointestinal bleeding or perforation, hyperglycaemia, hyperlipemia, osteoporosis and iatrogenic hyperadrenocorticism. Thus, in order to avoid adrenal insufficiency symptoms, the patient was administered methylprednisolone instead of prednisone during surgery (0.8 mg/kg/day, including the day before and after surgery).

**Fig. 2:**
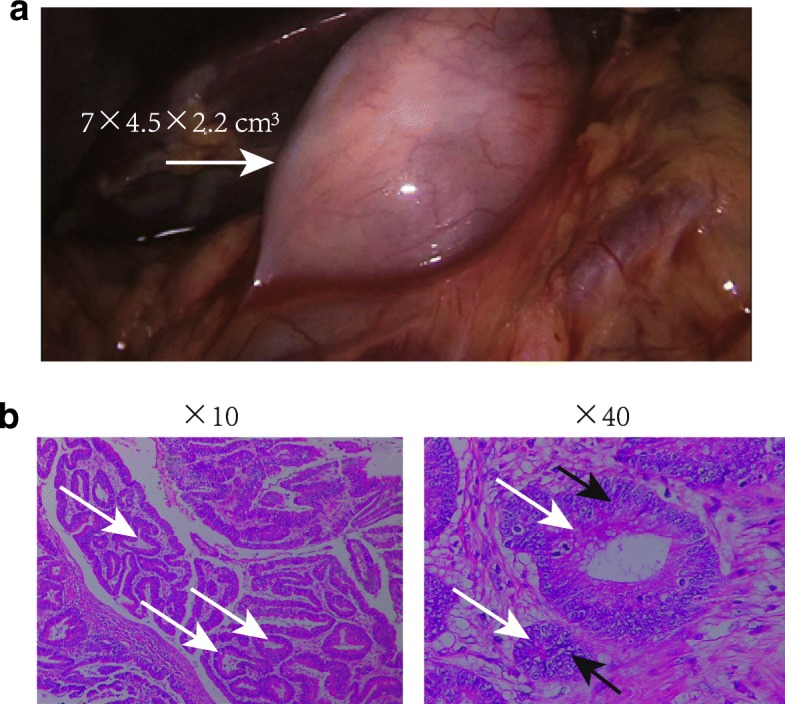
The morphology of tumor and the staining of tumor sections. a, a space-occupying lesion (white arrow) around the wall of the cecum during the laparoscopic surgery; b, HE staining of tumor sections. The tumor nests, white arrow; pleomorphic nuclei with clear, foamy, or vesicular cytoplasm, black arrow; HE, Hematoxylin and eosin

During the laparoscopic surgery, a huge and hard space-occupying lesion was observed around the wall of the cecum, extending into the serosa and retroperitoneum (Fig. 2a). The liver, stomach, duodenum and pelvic cavity appeared normal, and the pelvic cavity was negative for peritoneal fluid accumulation. A drainage tube was placed at anastomotic site without preventive colostomy because the tissues of patient were good in elasticity and the blood supply of ileum and transverse colon was normal.

The biopsy report indicated a moderately differentiated adenocarcinoma with mucinous differentiation (size: 7 × 4.5 × 2.2 cm^3^; Fig. 2a&b). The metastasis was only detected in the adjacent lymph node of the tumor (1/17). The tumor was classified as T3N1M0 (stage III).

The patient was subsequently treated by mFOLFOX6 chemotherapy (oxaliplatin/5-fu/leucovorin). The postoperative level of plasma glucose was carefully monitored. The patient was also treated with proton pump inhibitors post-surgery to prevent the development of stress ulcers. Postoperative calcium supplementation was also offered. Another postoperative consideration was that lipid emulsion was not used. The patient recovered well after the surgery and the chemotherapy (Tables 1 and 2).

**Table 2:** Timeline in this case report

Date	Information
Jun/19/2017	The patient was referred to our hospital
Jun/19-Jun/25/2017	Physical examination;
	Abdominal computed tomography scan and colonoscopy
Jun/27/2017	Pre-operative examination; Methylprednisolone (40 mg/d)
Jun/28/2017	Radical bowel resection; Methylprednisolone (40 mg/d)
Jun/29-Jul/1/2017	Post-operative observation; Methylprednisolone (40 mg/d)
Jul/28/2017	mFOLFOX6 chemotherapy

## Discussion and conclusions

Surgery for colon cancer in patients with SLE is difficult due to the concern of triggering adrenal insufficiency symptoms. With limited available reports on the use of chemotherapy after surgery for colon cancer in patients with SLE, we provide further evidence of its potential success.

Approximately 50% of patients with SLE present gastrointestinal symptoms [2]. According to this report, the clinical gastrointestinal symptoms in the patient with SLE and colon cancer showed limited specificity. Difficult defecation, watery stools and abdominal pain can be confounded in lupus-associated enteritis [1]. The causes of these clinical gastrointestinal symptoms are also multiple, including infection, medication side effects or simply a comorbid medical condition [1].

Table 3 summarized the information of four other patients with SLE and coexisting colorectal cancer [3–6]. The type of neoplasms in our patient and those in the case reports in Table 3 was adenocarcinoma. Only our report provided the data of the tumor biomarkers, carcinoembryonic antigen and CA19–9 [7], which was though not significantly elevated. Our report is also first to describe the details of the surgical preparations, e.g. selecting proper surgical approaches for the possible complications of the drug treatment for SLE, and reducing the potential adrenal insufficiency symptoms during and after surgery.

**Table 3:** Summary of Colorectal Cancer reported in patients with SLE

Reference	Age	Sex	Cancer	Topography of lesions
[3]	NC (32–63)	NC	Adenocarcinoma	Colon
[4]	39	Female	Adenocarcinoma	Rectum
[5]	17	Female	Adenocarcinoma	Colon (one in the sigmoid and three in the colon ascendens)
[6]	75	Male	Adenocarcinoma	Descending colon

*NC* not clear

The radical bowel resection is a preferred method to improve long-term survival of patients with colon cancer [8]. Before the surgery, an evaluation of the postoperative intestinal condition is necessary to prevent surgery-induced intestinal fistula. In this report, the patient has taken a long-term glucocorticoid treatment for SLE, which increases the susceptibility to infection, gastrointestinal bleeding, hyperglycaemia, hyperlipemia, osteoporosis and iatrogenic hyperadrenocorticism. However, sudden withdrawal of SLE medications to reduce the risk of anastomotic leakage might cause severe symptoms of adrenal insufficiency, such as nausea, vomiting, weakness, hypotension and shock. Thus, the patient in our report was administered methylprednisolone during the surgery instead of oral prednisone. In addition, potential postoperative surgical site infection, adrenal insufficiency and adrenal crisis were also taken into consideration.

In conclusion, reports on treating patients diagnosed with both SLE and colon cancer are extremely limited, and an established treatment strategy is lacking yet necessary. We demonstrated that surgical resection to treat colon cancer in patients with SLE has the potential to produce systematic complications, and pharmacological adverse effects must be strongly considered. In this report, we provided a reference for safely and effectively treating a patient with both colon cancer and SLE through radical resection and subsequent chemotherapy.

## Abbreviations

CT: Computed tomography
SLE: Systemic lupus erythematous

## References

[1] Brewer BN, Kamen DL (2018). Gastrointestinal and hepatic disease in systemic lupus erythematosus. Rheum Dis Clin N Am.

[2] Ebert EC, Hagspiel KD (2011). Gastrointestinal and hepatic manifestations of systemic lupus erythematosus. J Clin Gastroenterol.

[3] Lewis RB, Castor CW, Knisley RE, Bole GG (1976). Frequency of neoplasia in systemic lupus erythematosus and rheumatoid arthritis. Arthritis Rheum.

[4] Lopez Dupla M, Khamashta M, Pintado Garcia V, Lavilla Uriol P, Valencia Ortega E, Gil Aguado A (1993). Malignancy in systemic lupus erythematosus: a report of five cases in a series of 96 patients. Lupus.

[5] Rahner N, Höefler G, Högenauer C, Lackner C, Steinke V, Sengteller M, Friedl W, Aretz S, Propping P, Mangold E, Walldorf C (2008). Compound heterozygosity for two MSH6 mutations in a patient with early onset colorectal cancer, vitiligo and systemic lupus erythematosus. Am J Med Genet A.

[6] Kijima T, Kanekiyo S, Nakasuga C, Inoue Y, Shindo Y, Tsutsui M, Yoshino S, Kubo M, Yano M, Bimoto M, Kanda T, Hazama S, Oka M (2013). A case report of a patient with overlap syndrome systemic lupus erythematosus (SLE) and polymyositis (PM) whose condition improved following treatment for coexisting descending colon cancer. Gan To Kagaku Ryoho.

[7] Vukobrat-Bijedic Z, Husic-Selimovic A, Sofic A, Bijedic N, Bjelogrlic I, Gogov B, Mehmedovic A (2013). Cancer antigens (CEA and CA 19-9) as markers of advanced stage of colorectal carcinoma. Med Arch.

[8] Mukkai Krishnamurty D, Wise PE (2016). Importance of surgical margins in rectal cancer. J Surg Oncol.

